# Blocking c-Met signaling enhances bone morphogenetic protein-2-induced osteoblast differentiation

**DOI:** 10.1016/j.fob.2015.04.008

**Published:** 2015-04-20

**Authors:** Seiji Shibasaki, Sachie Kitano, Miki Karasaki, Sachi Tsunemi, Hajime Sano, Tsuyoshi Iwasaki

**Affiliations:** a General Education Center, Hyogo University of Health Sciences, 1-3-6 Minatojima, Chuo-ku, Kobe, Hyogo 650-8530, Japan; b Division of Rheumatology, Department of Internal Medicine, Hyogo College of Medicine, 1-1 Mukogawa-cho, Nishinomiya, Hyogo 663-8501, Japan; c Division of Pharmacotherapy, Department of Pharmacy, School of Pharmacy, Hyogo University of Health Sciences, 1-3-6 Minatojima, Chuo-ku, Kobe, Hyogo 650-8530, Japan

**Keywords:** ALP, alkaline phosphatase, BMP, bone morphogenetic protein, ERK, extracellular signal-regulated kinase, HGF, hepatocyte growth factor, MAPK, mitogen-activated protein kinase, MEK, MAPK/ERK kinase, PI3K, phosphatidylinositol-3-kinase, RA, rheumatoid arthritis, RT-PCR, reverse transcription-polymerase chain reaction, c-Met, Hepatocyte growth factor, Osteoblast, Rheumatoid arthritis

## Abstract

We previously demonstrated that blocking hepatocyte growth factor (HGF) receptor/c-Met signaling inhibited arthritis and articular bone destruction in mouse models of rheumatoid arthritis (RA). In the present study, we investigated the role of c-Met signaling in osteoblast differentiation using the C2C12 myoblast cell line derived from murine satellite cells and the MC3T3-E1 murine pre-osteoblast cell line. Osteoblast differentiation was induced by treatment with bone morphogenetic protein (BMP)-2 or osteoblast-inducer reagent in the presence or absence of either HGF antagonist (NK4) or c-Met inhibitor (SU11274). Osteoblast differentiation was confirmed by Runx2 expression, and alkaline phosphatase (ALP) and osteocalcin production by the cells. Production of ALP, osteocalcin and HGF was verified by enzyme-linked immunosorbent assay. Runx2 expression was confirmed by reverse transcription-PCR analysis. The phosphorylation status of ERK1/2, AKT, and Smads was determined by Western blot analysis. Both NK4 and SU11274 enhanced Runx2 expression, and ALP and osteocalcin production but suppressed HGF production in BMP-2-stimulated C2C12 cells. SU11274 also enhanced ALP and osteocalcin production in osteoblast-inducer reagent-stimulated MC3T3-E1 cells. SU11274 inhibited ERK1/2 and AKT phosphorylation in HGF-stimulated C2C12 cells. This result suggested that ERK and AKT were functional downstream of the c-Met signaling pathway. However, both mitogen-activated protein kinase/ERK kinase (MEK) and phosphatidylinositol 3-kinase (PI3K) inhibitor suppressed osteocalcin and HGF production in BMP-2-stimulated C2C12 cells. Furthermore, SU11274, MEK, and PI3K inhibitor suppressed Smad phosphorylation in BMP-2-stimulated C2C12 cells. These results indicate that although the c-Met-MEK-ERK-Smad and c-Met-PI3K-AKT-Smad signaling pathways positively regulate osteoblast differentiation, c-Met signaling negatively regulates osteoblast differentiation, independent of the MEK-ERK-Smad and PI3K-AKT-Smad pathways. Therefore, blocking c-Met signaling might serve as a therapeutic strategy for the repair of destructed bone in patients with RA.

## Highlights

• Role of c-Met signaling in osteoblast differentiation was investigated.
• Osteoblast differentiation was determined by ALP and osteocalcin production by C2C12 and MC3T3-E1 cells.
• c-Met signaling negatively regulates osteoblast differentiation.
• Blocking c-Met signaling might serve as a therapeutic strategy for rheumatoid arthritis.

## 1 Introduction

Patients with rheumatoid arthritis (RA) often have severe systemic bone loss and increased risk of fracture due to increased bone resorption, and decreased bone formation [1]. Receptor activator of nuclear factor kappa-B ligand (RANKL), secreted by synovial tissues, plays a critical role in osteoclastogenesis [2]. Synovial fibroblasts from patients with RA express RANKL [3]. RANKL is also expressed by T cells in the synovial tissues of RA patients [4]. Bone formation requires coordination between osteoblasts and osteoclasts. This coordination is mediated by multiple growth factors and cytokines [5]. The bone morphogenetic proteins (BMPs) are members of the transforming growth factor (TGF)-β superfamily, and they play a central role in bone formation [6]. BMPs are expressed preferentially in mesenchymal tissues prefiguring the future skeleton, developing bones, and differentiated chondrocytes and osteoblasts [7]. Tumor necrosis factor (TNF)-α is highly expressed in patients with RA, and it inhibits bone formation by affecting major osteoblast regulatory pathways [8,9].

Angiogenic growth factors such as fibroblast growth factor (FGF)-2 and FGF-4 [10,11], and vascular endothelial growth factor (VEGF) [12] act synergistically with BMP-2 to promote osteoblast differentiation. Conversely, hepatocyte growth factor (HGF), which is an angiogenic growth factor, has an inhibitory effect on osteoblast differentiation [13,14]. HGF enhances angiogenesis, and HGF receptor (c-Met)-mediated signaling events appear to induce synovial cell proliferations in RA. NK4 is a fragment of HGF that was constructed by proteolytic digestion, and it consists of 447 residues with a molecular weight of approximately 55–69 kDa. NK4 comprises the N-terminal hairpin and subsequent four-kringle domains of HGF, but lacks the 16 amino acids at the C-terminus of the α-chain and the whole β-chain. NK4 functions as an HGF antagonist by competitively binding to c-Met [15,16]. We previously demonstrated that the HGF antagonist, NK4, inhibits arthritis by suppressing angiogenesis and inflammatory cytokine production by CD4^+^ T cells in SKG mice, an animal model of RA. We also demonstrated that articular bone destruction is inhibited by NK4 treatment [17]. In the present study, we investigated the role of c-Met signaling in osteoblast differentiation using C2C12 myoblasts, a cell line derived from murine satellite cells and the MC3T3-E1 murine pre-osteoblast cell line [18,19].

## 2 Materials and methods

### 2.1 Cell cultures

The C2C12 murine myoblast cell line and the MC3T3-E1 murine pre-osteoblast cell line were purchased from the American Type Culture Collection (Manassas, VA, USA) [18,19]. C2C12 or MC3T3-E1 cells were grown in Dulbecco’s Modified Eagle’s Medium (DMEM; Sigma, St. Louis, MO, USA) containing 10% fetal bovine serum (FBS) and antibiotics (100 units/mL penicillin and 100 μg/mL streptomycin) at 37 °C under a humid atmosphere of 95% air/5% CO_2_.

### 2.2 Alkaline phosphatase (ALP) and osteocalcin assays

The ALP and osteocalcin assays were performed as described previously [20]. Briefly, C2C12 or MC3T3-E1 cells were seeded in 24-well tissue culture plates at a density of 1 × 10^5^/mL/well. C2C12 cells were cultured with BMP-2 (300 ng/mL; R&D systems, Minneapolis, MN, USA) and MC3T3-E1 cells were cultured with the osteoblast-inducer reagent (2% β-glycerophosphate, 0.2% hydrocortisone, and 1% ascorbic acid-2-phosphate; TaKaRa, Shiga, Japan) and ALP or osteocalcin activities in the culture supernatants were determined after 7 or 10 days of culture, respectively. C2C12 cells were preincubated for 72 h in the presence of either mitogen-activated protein kinase (MAPK)/extracellular signal-regulated kinase (ERK) (MEK) 1/2 inhibitor (PD98059) or phosphatidylinositol 3 kinase (PI3 K) inhibitor (Ly294002; Promega, Madison, WI, USA). After rigorous washing, the cells were stimulated with BMP-2 (300 ng/mL) for an additional 10 days, and the osteocalcin activities in the culture supernatants were determined. The concentrations of ALP or osteocalcin in the culture supernatants were determined using a mouse ALP (Bio Vision Research Products Mountain View, CA, USA) or osteocalcin (Biomedical Technologies, Inc., Stoughton, MA, USA) enzyme-linked immunosorbent assay (ELISA) kit, respectively.

### 2.3 HGF ELISA

The concentrations of HGF in the culture media were assayed using a mouse HGF ELISA kit (RayBio, Norcross, GA, USA).

### 2.4 Western blot analysis

Western blot analysis was performed as described previously [21]. Briefly, C2C12 cells were seeded in 12-well tissue culture plates at an initial density of 2 × 10^6^ cells/mL/well, and then stimulated with BMP-2 (300 ng/mL). After 10 min of stimulation, Western blot analysis was performed. Briefly, the cells were lysed in radio-immunoprecipitation assay (RIPA) lysis buffer (Santa Cruz Biotechnology, CA, USA), and the protein content was determined using Bio-Rad protein assay reagent (Bio-Rad, Hercules, CA, USA), with bovine serum albumin as the standard. Each sample (20 μg) was resolved on a 10 % polyacrylamide gel under denaturing conditions and then transferred to a 0.45-μm nitrocellulose membrane. After blocking overnight at 4 °C with 5% nonfat milk in Tris-buffered saline containing 0.01% Tween® 20 (Santa Cruz Biotechnology), the membranes were incubated overnight at 4 °C with anti-phospho-ERK 1/2 antibody (1:1000 dilution in phosphate-buffered saline [PBS]; Santa Cruz Biotechnology), anti-phospho-AKT antibody (1:1000 dilution in PBS; Santa Cruz Biotechnology), anti-phospho-smad1/5/8 antibody (1:1000 dilution in PBS; Santa Cruz Biotechnology), or mouse anti-β-actin antibody (Cell Signaling Technology, Beverly, MA, USA). After washing the membranes with Tris-buffered saline containing 0.05% Tween® 20 (washing buffer), horseradish peroxidase (HRP)-conjugated secondary antibody (1:1000 dilution in PBS; Santa Cruz Biotechnology) was added, followed by incubation for 45 min. After further washing, color was developed using luminol reagent (Santa Cruz Biotechnology), and the HRP activity of the blots was analyzed using a LAS1000 imager (Fuji film, Tokyo, Japan).

### 2.5 Quantitative reverse transcription-polymerase chain reaction (RT-PCR)

Runx2 mRNA expression was determined by using quantitative RT-PCR. C2C12 cells were seeded in 24-well tissue culture plates at a density of 1 × 10^6^ cells/mL/well. The cells were stimulated with BMP-2 (300 ng/mL). After 24 h of culture, RNA was extracted and quantitative RT-PCR was performed using a TaKaRa PCR kit (Takara). Primers used for Runx2 and β-actin RT-PCR assay were purchased from Applied Biosystems (Tokyo, Japan). Data represent the relative expression levels of Runx2 mRNA to control β-actin mRNA.

### 2.6 Statistical analysis

The results are expressed as the mean ± standard error (SE). The significance of the differences between the experimental results and the control values was determined by Student’s *t*-test. *p* values less than 0.05 were considered significant.

## 3 Results

### 3.1 HGF antagonist (NK4) and c-Met inhibitor (SU11274) enhance osteoblast differentiation by C2C12 cells

**Fig. 1:**
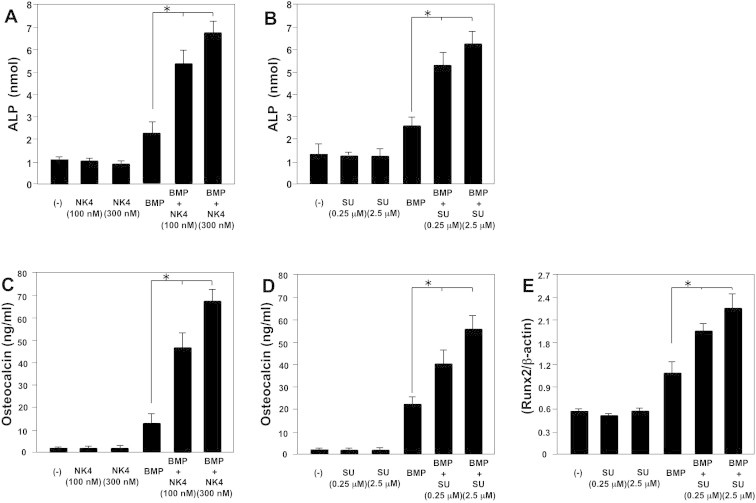
The hepatocyte growth factor (HGF) antagonist, NK4, and the c-Met inhibitor, SU11274, enhance osteoblast differentiation by C2C12 cells. C2C12 cells were treated with bone morphogenetic protein (BMP)-2 (300 ng/mL) with or without either NK4 (100–300 nM; Fig. 1A, C) or SU11274 (0.25–2.5 μM; Fig. 1B, D) at a density of 1 × 10^5^/mL/well and cultured for either 7 or 10 days. ALP production by the cells was determined after 7 days culture (Fig. 1A, B) and osteocalcin production by the cells was determined after 10 days culture (Fig. 1C, D). Runx2 mRNA expression was determined by using quantitative reverse transcription-polymerase chain reaction (RT-PCR). C2C12 cells were seeded in 24-well tissue culture plates at a density of 1 × 10^6^ cells/mL/well. The cells were stimulated with BMP-2 (300 ng/mL) with or without c-Met inhibitor. After 24 h of culture, RNA was extracted and quantitative RT-PCR was performed (Fig. 1E). Data represent relative expression of Runx2 mRNA to β-actin mRNA (control). Data are presented as mean ± standard error (SE; *n* = 3) for each experimental group. ^∗^*p* < 0.05 vs. BMP-2 treatment.

Using C2C12 myoblasts, we examined the effect of NK4 on osteoblast differentiation. First, we examined the effect of NK4 on the ALP activity of BMP-2-stimulated C2C12 cells. NK4 treatment enhanced ALP production in BMP-2-stimulated C2C12 cells after 7 days of culture (Fig. 1A). Osteocalcin is a late osteoblast differentiation marker [22]. We examined the effect of NK4 treatment on osteocalcin production by the cells. NK4 treatment enhanced osteocalcin production in BMP-2-stimulated C2C12 cells after 10 days of culture (Fig. 1C). To confirm the effects of c-Met signaling on osteoblast differentiation, we next examined the effects of c-Met inhibitor on osteoblast differentiation. c-Met inhibitor (SU11274) significantly enhanced both ALP and osteocalcin production in BMP-2-stimulated C2C12 cells (Fig. 1B, D). Runx2 is an osteoblast-specific transcription factor, which is essential for the differentiation of osteoblasts from mesenchymal precursors [23,24]. Therefore, we examined whether Runx2 expression in BMP-2-stimulated C2C12 cells was enhanced upon treatment with c-Met inhibitor. We examined the mRNA expression levels of Runx2 in BMP-2-stimulated C2C12 cells in the presence or absence of c-Met inhibitor (SU11274). After 24 h of culture in the presence of c-Met inhibitor, Runx2 mRNA levels were enhanced in BMP-2-stimulated C2C12 cells (Fig. 1E). These results indicate that blocking c-Met signaling in C2C12 cells enhances osteoblast differentiation.

### 3.2 Blocking c-Met signal suppresses HGF production by C2C12 cells

**Fig. 2:**
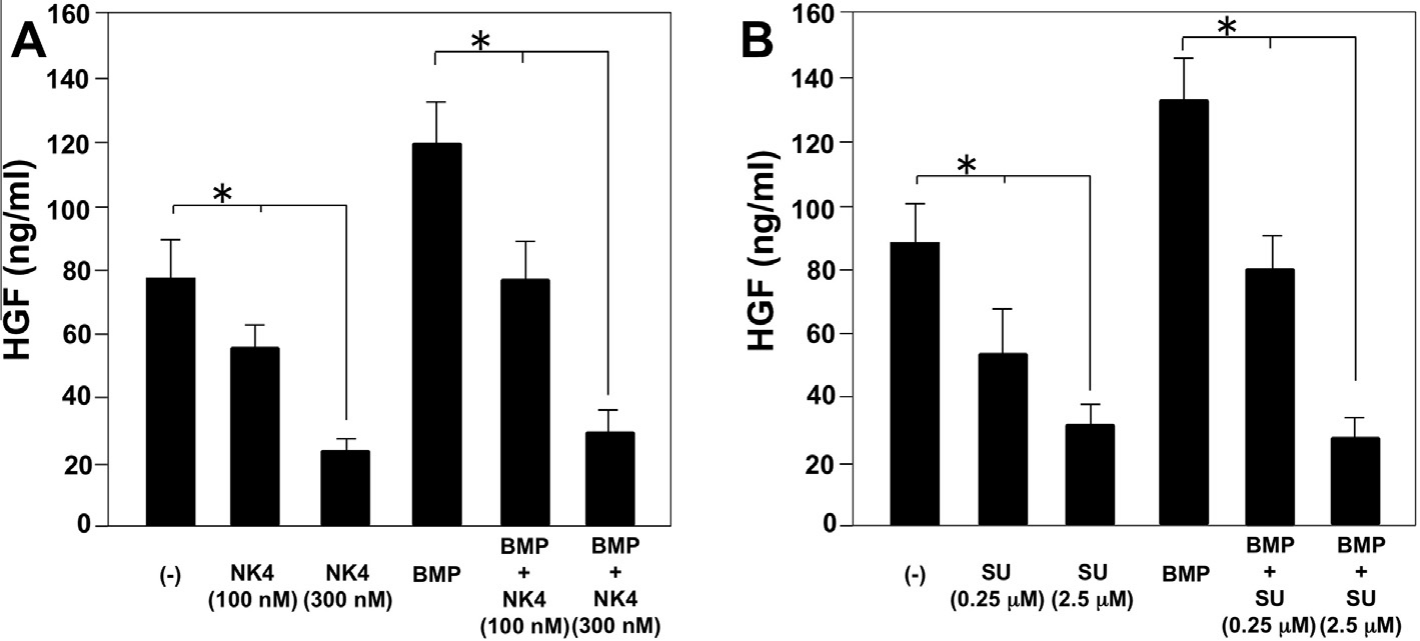
Blocking c-Met signaling inhibits HGF production by C2C12 cells. C2C12 cells were treated with BMP-2 (300 ng/mL) with or without either NK4 (100–300 Nm; Fig. 2A) or SU11274 (0.25–2.5 μM; Fig. 2B) at a density of 1 × 10^5^/mL/well and cultured for 4 days. HGF production by the cells was determined. Data are presented as mean ± SE (*n* = 3) for each experimental group. ^∗^*p* < 0.05 vs. control treatment.

C2C12 cells produce HGF, which in turn suppresses osteoblast differentiation. This indicates that HGF negatively regulates osteoblast differentiation [13,14]. We next examined the effect of blocking c-Met signaling on HGF production by C2C12 cells. C2C12 cells produced significant amounts of HGF, and BMP-2 treatment enhanced HGF production by C2C12 cells. Conversely, both NK4 (Fig. 2A) and SU11274 (Fig. 2B) suppressed HGF production by C2C12 cells. These results indicate that blocking c-Met signaling suppresses HGF production.

### 3.3 c-Met inhibitor (SU11274) enhances ALP and osteocalcin production in the pre-osteoblast cell line (MC3T3-E1 cells)

**Fig. 3:**
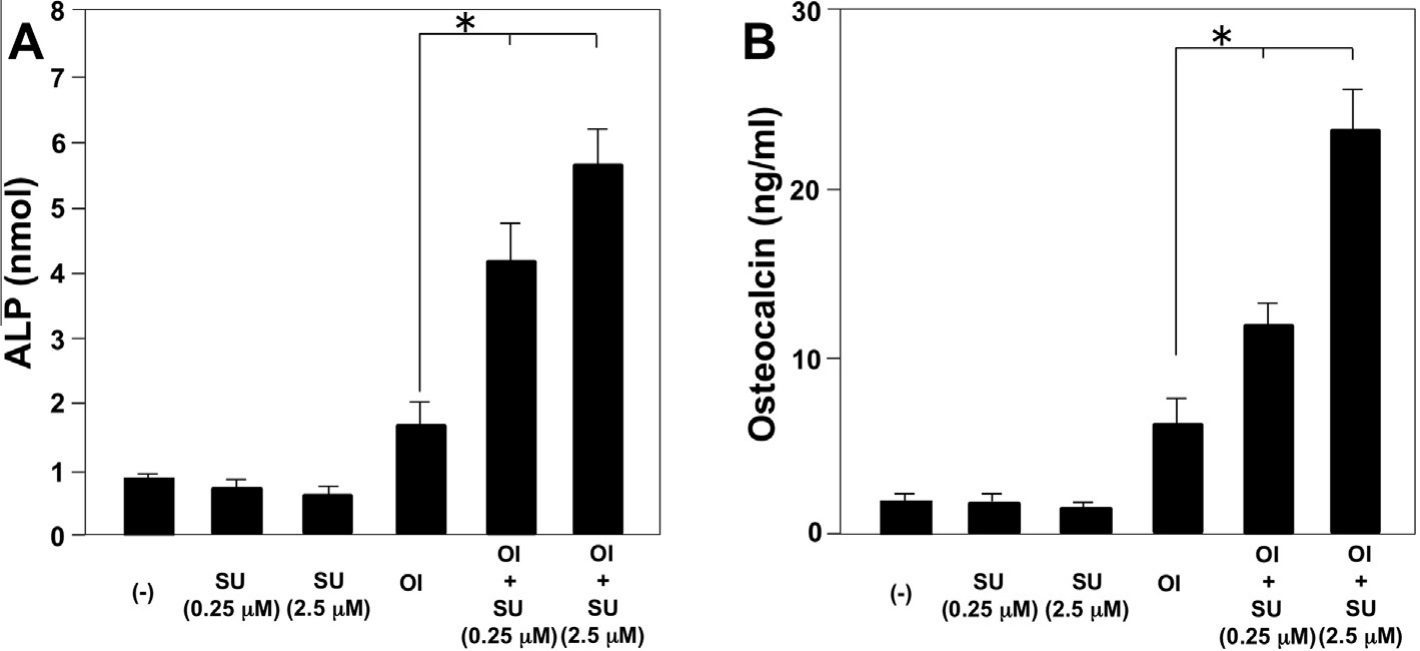
c-Met inhibitor (SU11274) enhance ALP and osteocalcin production by the pre-osteoblast cell line (MC3T3-E1 cells). MC3T3-E1 cells were seeded in 24-well tissue culture plates at a density of 1 × 10^5^/mL/well. Cells were cultured with osteoblast- inducer reagent (OI; 2% β-glycerophosphate, 0.2% hydrocortisone, and 1% ascorbic acid-2-phosphate) with or without c-Met inhibitor. ALP (Fig. 3A) or osteocalcin (Fig. 3B) activities in the culture supernatants were determined after 7 or 10 days of culture, respectively. Data are presented as mean ± SE (*n* = 3) for each experimental group. ^∗^*p* < 0.05 vs. control treatment.

We next examined the effect of c-Met inhibitor on the pre-osteoblast cell line (MC3T3-E1 cells). c-Met inhibitor (SU11274) significantly enhanced both ALP and osteocalcin production in osteoblast-inducer reagent-stimulated MC3T3-E1 cells (Fig. 3A, B).

### 3.4 SU11274 inhibits ERK1/2 and AKT phosphorylation by C2C12 cells

**Fig. 4:**
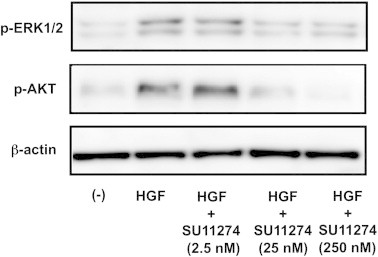
SU11274 inhibits ERK1/2 and AKT phosphorylation in C2C12 cells. C2C12 cells were treated with HGF (10 ng/mL) with or without SU11274 (2.5–250 nM) and p-ERK1/2 and p-AKT expression was determined by Western blot analysis. β-Actin expression was used as a control. Representative data for p-ERK1/2 and p-AKT expression in C2C12 cells are shown.

The MEK/ERK and PI3-AKT signaling pathways can be activated by a variety of growth factors such as insulin and HGF [25–28]. To determine whether ERK and AKT functioned downstream of the c-Met signaling pathway, we examined the effects of SU11274 on ERK1/2 and AKT phosphorylation in response to HGF treatment. Stimulation of C2C12 cells with HGF led to a significant increase in the phosphorylation levels of ERK1/2 and AKT. Treatment with c-Met inhibitor reduced HGF-mediated ERK1/2 and AKT phosphorylation (Fig. 4). These results indicate that the c-Met-MEK-ERK and c-Met-PI3-AKT signaling pathways are active in C2C12 cells.

### 3.5 Effect of MEK 1/2 or PI3K inhibitor on osteoblast differentiation

**Fig. 5:**
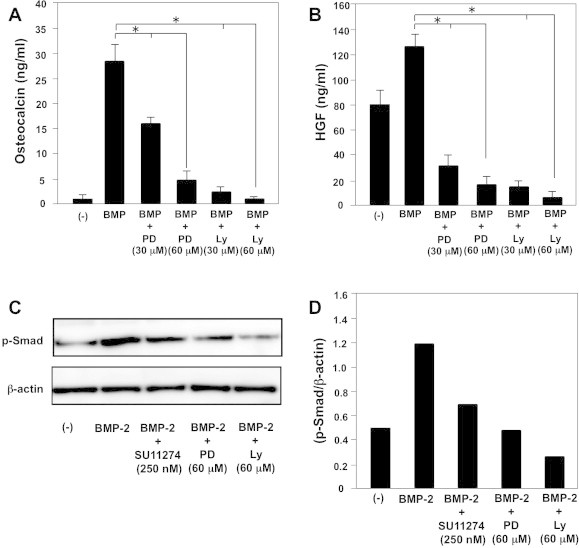
Effect of mitogen-activated protein kinase (MAPK)/ERK kinase (MEK) 1/2 or PI3 K inhibitor on osteoblast differentiation and p-Smads induction. C2C12 cells were treated with BMP-2 (300 ng/mL) with or without either PD98059 (60 μM) or Ly294002 (60 μM) at a density of 1 × 10^5^/mL/well and cultured for 10 days. Osteocalcin (Fig. 5A) or HGF (Fig. 5B) production by the cells was determined. Data are presented as mean ± SE (*n* = 3) for each experimental group. ^∗^*p* < 0.05 vs. BMP-2 treatment. C2C12 cells were treated with BMP-2 (300 ng/mL) with or without either SU11274 (250 nM), PD98059 (60 μM), or Ly294002 (60 μM) for 10 min and p-Smad1/5/8 expression was determined by Western blot analysis. Representative data for p-Smad1/5/8 and β-actin expression by C2C12 cells (Fig. 5C). Relative p-Smad1/5/8 expression (p-Smad1/5/8/β-actin; Fig. 5D).

We observed that blocking c-Met signaling enhanced ALP and osteocalcin production by BMP-2-stimulated C2C12 cells (Fig. 1) and that the c-Met-PI3-AKT and c-Met-MEK-ERK signaling pathways are active in C2C12 cells (Fig. 4). Therefore, we examined the effects of MEK1/2 inhibitor (PD98059) or PI3 K inhibitor (Ly294002) on osteocalcin production by BMP-2-stimulated C2C12 cells. In contrast to c-Met inhibitor, both PD98059 and Ly294002 significantly suppressed osteocalcin production by C2C12 cells (Fig. 5A). PD98059 and Ly294002 also suppressed HGF production by C2C12 cells (Fig. 5B). These results indicate that blocking both c-Met signaling and other signaling pathways downstream of c-Met such as MEK-ERK and PI3-AKT suppresses HGF production. However, blocking only the MEK-ERK and PI3-AKT pathways suppressed osteoblast differentiation by C2C12 cells.

### 3.6 SU11274 inhibits Smad induction by C2C12 cells

The osteogenic activity of BMP-2 is partly mediated by nuclear phosphorylation and nuclear translocation of Smads, which interact directly with DNA and associate with other transcription factors to regulate osteogenesis [23,29]. Therefore, we investigated whether blocking c-Met signaling enhanced osteoblast differentiation by altering the phosphorylation status of Smads. Because the PI3-AKT and MEK-ERK signaling pathways function downstream of the c-Met signaling pathway in C2C12 cells, we also examined the effect of MEK1/2 inhibitor (PD98059) or PI3 K inhibitor (Ly294002) on the phosphorylation status of Smads. BMP-2 enhanced Smad phosphorylation in C2C12 cells. SU11274 inhibited BMP-2-stimulated Smad phosphorylation in C2C12 cells. In addition, both PD98059 and Ly294002 significantly inhibited BMP-2-stimulated Smad phosphorylation in C2C12 cells (Fig. 5C, D). Taken together these results indicate that the c-Met-PI3-AKT-Smad and c-Met-MEK-ERK-Smad signaling pathways positively regulate osteoblast differentiation. However, independent of the MEK-ERK-Smad and PI3 K-AKT-Smad pathways, c-Met signaling negatively regulates osteoblast differentiation.

## 4 Discussion

We previously reported that the HGF antagonist, NK4, inhibited arthritis and bone destruction by inhibiting angiogenesis and inflammatory cell infiltration in the synovium in SKG mice [15]. In this study, we demonstrated that blocking c-Met signaling induces bone formation by enhancing osteoblast differentiation in C2C12 myoblasts. Blocking c-Met signaling by HGF antagonist (NK4) or c-Met inhibitor (SU11274) enhanced osteocalcin production by C2C12 cells stimulated with BMP-2 (Fig. 1). C2C12 cells spontaneously produced significant amounts of HGF, which inhibited BMP-induced osteoblast differentiation [13,14]. Although BMP-2 stimulation enhanced HGF production, both NK4 and SU11274 inhibited BMP-2-induced HGF production by C2C12 cells (Fig. 2). These results suggest that both NK4 and SU11274 directly block c-Met signaling not only by binding to its receptors but also by suppressing HGF production, which negatively regulates osteoblast differentiation by C2C12 cells.

To confirm that both ERK1/2 and AKT are downstream signal molecules of c-Met we examined the phosphorylation status of these molecules after stimulation with HGF. Inhibition of c-Met signaling using c-Met inhibitor suppressed both ERK1/2 and AKT phosphorylation by HGF-stimulated C2C12 cells, suggesting that the c-Met-MEK-ERK and c-Met-PI3K-AKT signaling pathways are active in C2C12 cells (Fig. 4). However, in contrast to c-Met inhibitor, inhibitors of both MEK1 and PI3K suppressed osteocalcin production in BMP-2-stimulated C2C12 cells (Fig. 5A). These results suggest that the c-Met-MEK-ERK and c-Met-PI3K-AKT pathways positively regulate osteoblast differentiation, but c-Met signaling negatively regulates osteoblast differentiation, independent of the MEK-ERK and PI3K-AKT pathways.

BMP receptors are serine/threonine kinase receptors and they may be classified into two types: I (BMPR-I) and II (BMPR-II). After BMP binding, BMPR-I kinases are activated by BMPR-II kinase-induced phosphorylation. Smad proteins are then required to activate the receptors. Therefore, Smads play a role in transmitting the BMP signal from the receptor to the target gene [23,29]. In addition to c-Met inhibitor, inhibitors of MEK1 and PI3K also suppressed BMP-2-induced Smad phosphorylation in C2C12 cells, suggesting that the c-Met-MEK-ERK and c-Met-PI3K-AKT signaling pathways positively regulate BMP-2-induced Smad phosphorylation (Fig. 5C, D).

In addition to the activation of Smads, BMP-2 activates non-Smad signaling molecules, including members of the MAPK family such as p38, ERK1/2, and JNK [30–32]. Furthermore, it was demonstrated that PI3K and its downstream target, AKT, are required for the BMP-2-induced expression of an osteoblast differentiation marker, alkaline phosphatase, and for BMP-2 transcription [33,34]. Therefore, BMP-2-induced MEK-ERK and PI3K-AKT signaling pathways may also induce osteoblast differentiation by C2C12 cells, independent of the Smad signaling pathways. Our results indicate that although both c-Met-MEK-ERK-Smad and c-Met-PI3K-AKT-Smad signals enhance osteoblast differentiation, c-Met signaling, independent of MEK-ERK-Smad and PI3K-AKT-Smad signaling, strongly suppresses osteoblast differentiation in C2C12 cells.

Several studies have demonstrated that c-Met signaling stimulates osteoblastic differentiation in several cell types. Aenlle et al. demonstrated that HGF promote osteoblast differentiation by inducing rapid phosphorylation of p38 using human mesenchymal stem cells [35]. Chen et al. demonstrated that HGF in combination with a known inducer of osteogenic differentiation, 1,25-dihydroxyvitamin D, significantly increased osteoblast differentiation in human bone marrow-derived stem cells [36]. However, our studies suggest that the HGF antagonist promotes osteoblast differentiation. Why these studies yielded contradictory results in different cell types is not clear. One reason for this discrepancy may be the different cell sources that were used in the studies. We used murine cell line such as C2C12 and MC3T3-E1, while Aenlle et al. and Chen et al. used human cells in their experiments. Another possible reason is the difference in the timing of HGF treatment. Kawasaki et al. reported that treatment with HGF during BMP-2-induced osteoblast differentiation enhanced osteoblast differentiation. In contrast, they also demonstrated that treatment of HGF prior to BMP-2 induced cellular proliferation did not influence subsequent osteoblast differentiation [14].

To our knowledge, this is the first study to demonstrate that blocking c-Met signaling enhances osteoblast differentiation, independent of the MEK-ERK-Smad and PI3K-AKT-Smad signaling pathways. Severe articular destruction in RA patients is caused not only by synovial cell proliferation (the synovial cells invade articular bone) but also by severe local osteoporosis due to inflammatory reactions. Therefore, Inhibition of c-Met signaling might be a useful therapeutic strategy for the treatment of RA by enhancing osteoblast differentiation in patients with RA.
